# Global economic productivity losses from vision impairment and blindness

**DOI:** 10.1016/j.eclinm.2021.100852

**Published:** 2021-04-26

**Authors:** Ana Patricia Marques, Jacqueline Ramke, John Cairns, Thomas Butt, Justine H. Zhang, Debbie Muirhead, Iain Jones, Brandon A.M.Ah Tong, Bonnielin K Swenor, Hannah Faal, Rupert R.A. Bourne, Kevin D. Frick, Matthew J. Burton

**Affiliations:** a London School of Hygiene & Tropical Medicine, London WC1E 7HT, United Kingdom; b School of Optometry and Vision Science, University of Auckland, Auckland, New Zealand; c University College London, London, United Kingdom; d Manchester Royal Eye Hospital, Manchester, United Kingdom; e The Fred Hollows Foundation, Melbourne, Australia; f Nossal Institute for Global Health, Melbourne School of Population and Global Health, University of Melbourne, Melbourne, VIC, Australia; g Sightsavers, Haywards Heath, United Kingdom; h The Wilmer Eye Institute, Johns Hopkins University, Baltimore, United States; i Department of Epidemiology, Johns Hopkins Bloomberg School of Public Health, Baltimore, United States; j Department of Ophthalmology, University of Calabar, Calabar, Nigeria; k Africa Vision Research Institute, Durban, Kwa-Zulu Natal, South Africa; l Vision & Eye Research Institute, Anglia Ruskin University, Cambridge, United Kingdom; m Department of Ophthalmology, Cambridge University Hospitals, Cambridge, United Kingdom; n Carey Business School, Johns Hopkins University, Baltimore, United States; o Moorfields Eye Hospital NHS Foundation Trust, London, United Kingdom

## Abstract

**Background:**

In the absence of accessible, good quality eye health services and inclusive environments, vision loss can impact individuals, households and communities in many ways, including through increased poverty, reduced quality of life and reduced employment. We aimed to estimate the annual potential productivity losses associated with reduced employment due to blindness and moderate and severe vision impairment (MSVI) at a regional and global level.

**Methods:**

We constructed a model using the most recent economic, demographic (2018) and prevalence (2020) data. Calculations were limited to the working age population (15–64 years) and presented in 2018 US Dollars purchasing power parity (ppp). Two separate models, using Gross Domestic Product (GDP) and Gross National Income (GNI), were calculated to maximise comparability with previous estimates.

**Findings:**

We found that 160.7 million people with MSVI or blindness were within the working age and estimated that the overall relative reduction in employment by people with vision loss was 30.2%. Globally, using GDP we estimated that the annual cost of potential productivity losses of MSVI and blindness was $410.7 billion ppp (range $322.1 - $518.7 billion), or 0.3% of GDP. Using GNI, overall productivity losses were estimated at $408.5 billion ppp (range $320.4 - $515.9 billion), 0.5% lower than estimates using GDP.

**Interpretation:**

These findings support the view that blindness and MSVI are associated with a large economic impact worldwide. Reducing and preventing vision loss and developing and implementing strategies to help visually impaired people to find and keep employment may result in significant productivity gains

**Funding:**

MJB is supported by the Wellcome Trust (207472/Z/17/Z). JR's appointment at the University of Auckland is funded by the Buchanan Charitable Foundation, New Zealand. The *Lancet Global Health* Commission on Global Eye Health was supported by grants from The Queen Elizabeth Diamond Jubilee Trust, Moorfields Eye Charity (GR001061), NIHR Moorfields Biomedical Research Centre, The Wellcome Trust, Sightsavers, The Fred Hollows Foundation, The SEVA Foundation, The British Council for the Prevention of Blindness and Christian Blind Mission. The funders had no role in the design, conduct, data analysis of the study, or writing of the manuscript.

**Research in context**

**Evidence before this study:**

We conducted a systematic review to describe and summarize the costs associated with vision impairment and its major causes at a global level (reported elsewhere). In brief, a literature search (2000–2019) with no geographic or language restrictions was performed in MEDLINE (Ovid) and the CRD database (Centre for Reviews and Dissemination) in December 2019. Only three studies reported productivity loss estimates at a global or multi-region level for blindness and vision impairment. The widespread use of assumptions to produce productivity loss estimates in many studies highlighted the lack of reliable and up-to-date data sources for most regions. These older estimates, based on outdated data, less robust information for parameters, and little assessment of uncertainty, have limitations in terms of reliability and current applicability.

**Added value of this study:**

As part of the *Lancet Global Health* Commission on Global Eye Health, this economic modelling study uses the most recent economic, demographic and prevalence data on moderate and severe vision impairment (MSVI) and blindness to estimate the annual cost of potential productivity losses due to unaddressed blindness and MSVI globally and for each Global Burden of Disease (GBD) region. Further, we based our estimates of the relative reduction in employment due to vision loss on a literature review, instead of following the assumptions made in previous studies. We estimated that the annual global cost in potential productivity losses due to blindness and MSVI was approximately $410.7 billion ppp (range $322.1 to $518.7 billion) in 2018.

**Implications of all the available evidence:**

Our findings support the view that blindness and MSVI are associated with a large economic impact worldwide. All regions of the world could achieve significant productivity gains if eye health services were more accessible, and included prevention and treatment of vision loss as well as comprehensive rehabilitation services. It is also critical to implement strategies to enable visually impaired people to find and keep employment, and create more accessible and inclusive cultures and environments for people with vision loss.

Alt-text: Unlabelled box

## 1 Introduction

Worldwide in 2020 an estimated 596.2 million people have distance vision impairment, of whom 43.3 million are blind and 295.1 million have moderate or severe vision impairment (MSVI) [1]. A further 509.7 million have uncorrected near vision impairment. Vision impairment and impaired eye health can have a wide-reaching and major impact on the lives of individuals, their families and society [2]. Vision impairment can cause or exacerbate poverty through reduced employment prospects and work productivity [3], [4], [5], [6], as well as adversely affect educational opportunities and outcomes [7]. Impaired vision and eye health can also impact general health and well-being, with associated reductions in quality of life [8]. Therefore, eye health can be considered a broad-based development issue. Addressing population eye health and vision impairment has the potential to be a powerful enabler for achieving the Sustainable Development Goals (SDGs) [9,10]

Economic productivity at the individual, family and national level is critically important to sustainable development. From an economic perspective, the productive capacity of the economy is reduced when labour input (workforce) decreases through people being unemployed or underemployed. This is quantified by estimating productivity losses [11]. Illness and disability can contribute to productivity losses through one or more of: (1) an absence from work (absenteeism), (2) a reduction in production while at work (presenteeism), or (3) a reduction in employment including job loss and early retirement.

To build a more complete picture of the individual and societal impact of vision impairment, it is necessary to understand the extent of the associated attributable economic productivity losses. Combining this with other sources of evidence about the impact of vision impairment informs policy makers about the relative importance of eye health, and the potential costs and benefits of addressing this. As part of the *Lancet Global Health* Commission on Global Eye Health [12], in this study we estimated the annual economic productivity losses associated with reduced employment due to blindness and MSVI.

## 2 Methods

This study modelled the annual cost of productivity losses associated with reduced employment due to unaddressed blindness and MSVI globally and for each Global Burden of Disease (GBD) region. The calculation included: (1) the number of people with blindness or MSVI of working age (15–64 years) in 2020, (2) the employment-to-population ratio in 2018, (3) the relative reduction in employment for people with vision loss, and (4) per capita Gross Domestic Product (GDP) or Gross National Income (GNI) for 2018.

### 2.1 Prevalence of blindness and MSVI in the working age population

Blindness was defined as presenting distance visual acuity <3/60 in the better eye and MSVI as presenting distance visual acuity (i.e. with correction if usually worn) of between <6/18 to 3/60 in the better eye. As such, monocular blindness or MSVI were not included in the prevalence data. The working age population was defined as those aged 15 to 64 years old inclusive [13]. Data on the number of people of working age with blindness or MSVI (and 95% uncertainty intervals [UI]) in each GBD region in 2020 were provided by the GBD Study / Vision Loss Expert Group (VLEG) [1]. RB provided VLEG prevalence data in 5-year increments of age; from these data, APM extracted the working age population data by region. APM and RB had access to these data for analysis throughout the study period.

### 2.2 Employment-to-population ratio

We defined employment-to population ratio as the proportion of a country's population aged 15 years and over that is employed, in paid full-time or part-time employment or self-employment either at work or having a job but in temporary absence (e.g. parental leave, sick leave, annual leave) [14]. It is generally measured during a specified brief period, such as one week or one day. We sourced data from the World Bank´s World Development Indicator database for 2018, or the most recent year available (data were unavailable from 11 countries). [15]. APM collected these data and summarised them at a regional level (shown in supplementary Table 1).

### 2.3 Relative reduction in employment for people with vision loss

We estimated the relative reduction in employment by comparing levels of reported employment levels in people with and without vision loss. We searched for relevant literature in Medline (OVID) and Google in February 2020 using the search terms: (employment OR absenteeism OR presenteeism OR sick leave) AND (vision impairment OR visual impairment OR blindness OR cataract OR glaucoma OR age-related macular degeneration OR diabetic retinopathy). We sought studies or reports from any country published since the year 2000 that reported the employment status of people with vision loss and/or the employment reduction between people with and without vision loss.

We identified 11 peer-reviewed published studies [16], [17], [18], [19], [20], [21], [22], [23], [24], [25], [26] and five grey literature reports [27], [28], [29], [30], [31] that provided employment reduction data for 15 countries and WHO Mortality Stratum regions, which provide estimates for eight GBD regions and three GBD super regions (supplementary Table 2). Employment reduction was reported using employment rates or labour force participation rates. Many of these studies did not report how employment was defined and those which did used several different definitions (for example self-employment was not always included). Further, there was a range of definitions for vision loss and for the working age population. Employment data on people with vision loss were compared to either people without vision loss, people without any disability, or with the general population. The relative reduction in employment for each region and super region was calculated as the weighted average employment gap of the countries that reported data within each region or super region (with the total population of each country being the weight). Due to limited data, we could not disaggregate reduction in employment for blindness compared to MSVI, or for different age groups or separately for women and men. When estimating productivity losses by GBD region, we used the GBD super region average whenever there was no data for a specific region. If there were no data for both a region and its super region the global average of all super regions was used.

### 2.4 Gross domestic product (GDP) and gross national income (GNI)

We assumed the annual cost of potential productivity losses associated with reduced employment due to MSVI or blindness was equal to GDP or GNI per capita. GDP is the sum of the gross value added by all resident producers in the economy[32]. GNI is the sum of value added by all resident producers plus net receipts of primary income (compensation of employees and property income) from abroad [33]. We developed both GDP and GNI models to generate results that could be compared to previous estimates, as both approaches have been used in the past [16,34,35]. Data were sourced from the World Bank´s World Development Indicator database in 2018 US Dollar purchasing power parity (ppp) for 2018, or the most recent year for which data were available (summarised in supplementary Table 1; data were unavailable for six countries).

### 2.5 Estimating the annual cost of economic productivity loss

The annual potential productivity loss associated with reduced employment was estimated for each region, following an approach used several times previously[16,36,37] and using the formula:

$$\begin{array}{c} Annual\;potential\;productivity\;losses_{\left(region\;a\right)}=\;\\ Prev.\;Blindness\;and\;MSVI\;working\;age\;population_{\left(region\;a\right)}\;X\\ Employment-to-population-ratio{\;}_{\left(region\;a\right)}\;X\\ Relative\;Reduction\;in\;employment_{\left(region\;a\right)\;}\;X\\ \left[GDP\;per\;capita_{\left(region\;a\right)}\;or\;GNI\;per\;capita_{\left(region\;a\right)}\right] \end{array}$$

The employment-to-population ratio, GDP per capita ppp and GNI per capita ppp for each GBD region were calculated as the weighted average of the data from each country in the region with available data; the total population of each country was used as the weight (supplementary appendix) [38]. Similarly, the relative reduction in employment of people with vision loss for each region or super region was calculated as the weighted average reduction in employment for the countries in the region or super region using total population of each region country as the weight (supplementary appendix). Productivity losses by region are reported in billion 2018 US Dollars ($) ppp, and as a percentage of GDP ($ ppp) [39]. GDP ppp per region was calculated as the sum of GDP ppp of the countries included in each GBD region.

Sensitivity analyses were performed to evaluate whether the uncertainty of the prevalence data and the relative reduction in employment data result in substantive changes in the estimates. We used available published prevalence [1] and relative reduction in employment data for the sensitivity analysis [40], [41], [42]. First, the upper and lower values of the 95% uncertainty intervals of the number of people in the working age population with MSVI or blindness were used to generate a range for each of the productivity loss estimates. This range is presented with the global estimate and for the estimate calculated for each region. Second, we substituted the data on relative reduction in employment derived from the literature search with data from the Eurostat disability statistics and recalculated all estimates (supplementary Table 3) [40]. Eurostat disability statistics reported employment reduction data from 31 countries included in four regions and three super regions for people reporting disabilities in basic activities, defined as an ‘activity difficulty such as sight, hearing, walking and communicating’. Third, we substituted the data on the relative reduction in employment with the disability weights for blindness and vision impairment published by WHO. [41,42]. By doing so we used disability weights as a proxy for the extent of lost productivity assuming a linear relationship between productivity and disability weights. Disability weights for distance vision impairment are reported for four levels of severity (mild, moderate, severe and blindness) which did not align with the categories of prevalence data in our model (blindness and MSVI). Therefore, MSVI prevalence data had to be split, and we assumed an equal split between the moderate and severe categories.

### 2.6 Role of the funding source

The funders had no role in the study design, data collection, data analysis, data interpretation, writing of the manuscript, or in the decision to submit the manuscript for publication. This modelling study used published or publicly available data. APM and TB had full access to all the data in the study and all authors accept responsibility to submit for publication.

## 3 Results

Globally, in 2020, there were an estimated 18.1 million (95%UI 14.4 million - 22.6 million) people in the working age population who were blind and 142.6 million (95%UI 112.5 million - 179.5 million) who had MSVI. These people represent 41.9% and 48.3% of all people with blindness and MSVI, and 0.4% and 2.4% of the global working age population, respectively. The numbers of people affected in each region are presented in Table 1. The global average relative reduction in employment of people with vision impairment or blindness was estimated to be 30.2%. The available regional or super regional values are presented in Table 2.

**Table 1:** Blindness or moderate to severe vision impairment in the working age population (15–64 years) in the 21 Global Burden of Disease Study regions in 2020.

GBD regions	Blindness in the working age population			Moderate and severe vision impairment in the working age population		
	Number in millions (95%UI)	% Total Blindness (95%UI)	% Working age population (95% UI)	Number in millions (95%UI)	% Total MSVI (95%UI)	% Working age population (95% UI)
High-income Asia Pacific	0.17 (0.14–0.21)	31.6% (31.4%−31.7%)	0.1% (0.1%−0.2%)	1.82 (1.42–2.30)	34.1% (33.4%−34.5%)	1.5% (1.2% −2.0%)
Australasia	0.02 (0.02–0.03)	32.5% (32.0%−32.9%)	0.1% (0.1%−0.1%)	0.3 (0.24–0.39)	40.3% (39.9%−40.7%)	1.6% (1.2% −2.0%)
Western Europe	0.40 (0.31–0.51)	26.4% (26.1%−26.8%)	0.1% (0.1%−0.2%)	5.94 (4.62–7.52)	38.5% (37.9%−39.1%)	2.1% (1.7% −2.7%)
Southern Latin America	0.06 (0.05–0.08)	37.9% (37.7%−38.4%)	0.1% (0.1%−0.2%)	1.02 (0.79–1.29)	48.1% (47.5%−48.6%)	2.3% (1.8%−3.0%)
High-income North America	0.23 (0.18–0.29)	32.4% (32.1%−32.7%)	0.1% (0.1%−0.1%)	3.26 (2.54–4.11)	43.8% (43.2%−44.2%)	1.4% (1.2% −1.7%)
Central Asia	0.16 (0.12–0.21)	52.4% (52.4%−52.8%)	0.3% (0.2%−0.3%)	1.66 (0.30–2.10)	56.1% (55.6%−56.8%)	2.7% (2.1%−3.5%)
Central Europe	0.11 (0.09–0.14)	34.2% (34.1%−34.3%)	0.2% (0.1%−0.2%)	1.45 (1.12–1.87)	36.7% (36.1%−37.4%)	1.9% (1.5%−2.5%)
Eastern Europe	0.26 (0.21–0.33)	33.6% (33.4%−33.7%)	0.2% (0.2%−0.2%)	4.74 (3.69–6.06)	42.8% (42.0%−43.6%)	3.4% (2.6%−4.3%)
Caribbean	0.11 (0.08–0.14)	40.6% (40.5%−40.9%)	0.4% (0.3%−0.5%)	0.78 (0.61–0.99)	50.5% (49.8%−51.1%)	2.8% (2.1%−3.5%)
Andean Latin America	0.13 (0.10–0.17)	37.5% (37.4%−37.9%)	0.3% (0.3%−0.4%)	1.42 (0.10–1.79)	51.4% (50.4%−52.0%)	3.6% (2.8%−4.6%)
Central Latin America	0.54 (0.42–0.69)	42.8% (42.7%−43.1%)	0.3% (0.3%−0.4%)	5.29 (4.10–6.71)	53.7% (52.9%−54.4%)	3.2% (2.5%−4.0%)
Tropical Latin America	0.71 (0.57–0.87)	39.9% (39.8%−40.1%)	0.5% (0.4%−0.6%)	5.83 (4.54–7.34)	56.4% (55.7%−56.9%)	3.9% (3.0%−4.9%)
North Africa and Middle East	1.30 (0.99–1.71)	42.2% (42.0%−42.4%)	0.3% (0.3%−0.4%)	11.55 (9.15–14.44)	52.9% (52.5%−53.3%)	3.0% (2.4%−3.8%)
South Asia	4.92 (3.87–6.18)	41.2% (41.1%−41.5%)	0.4% (0.3%−0.5%)	50.68 (39.87–64.1)	52.7% (51.6%−53.9%)	4.4% (3.4%−5.5%)
Southeast Asia	2.67 (2.12–3.32)	44.8% (44.7%−45.0%)	0.6% (0.5%−0.7%)	13.88 (11.30–17.01)	48.2% (47.2%−49.3%)	3.1% (2.4%−3.7%)
East Asia	3.76 (3.02–4.64)	41.4% (40.9%−41.9%)	0.4% (0.3%−0.5%)	21.65 (16.8–27.78)	40.2% (39.4%−41.1%)	2.1% (1.7% −2.8%)
Oceania	0.02 (0.02–0.03)	62.4% (62.3%−62.9%)	0.4% (0.3%−0.5%)	0.22 (0.17–0.28)	56.8% (56.2%−57.5%)	3.3% (2.6% −4.2%)
Central Sub-Saharan Africa	0.17 (0.12–0.22)	57.5% (57.4%−58.3%)	0.3% (0.2%−0.3%)	1.13 (0.88–1.45)	56.4% (56.0%−56.8%)	1.7% (1.3% −2.2%)
Eastern Sub-Saharan Africa	1.03 (0.81–1.3)	52.3% (52.2%−52.4%)	0.5% (0.4%−0.6%)	3.81 (3.01–4.79)	54.4% (53.9%−54.8%)	1.8% (1.4% −2.2%)
Southern Sub-Saharan Africa	0.22 (0.18–0.28)	46.3% (46.2%−46.4%)	0.4% (0.4%−0.6%)	0.84 (0.65–1.06)	53.6% (53.1%−54.1%)	1.7% (1.3% −2.1%)
Western Sub-Saharan Africa	1.12 (0.88–1.42)	47.8% (47.8%−48.3%)	0.5% (0.4%−0.6%)	5.35 (4.22–6.75)	54.3% (53.7%−54.9%)	2.4% (1.9% −3.0%)
**Total**	**18.12 (14.42–22.62)**	**41.9% (41.8%−42.1%)**	**0.4% (0.3%−0.5%)**	**142.62 (112.50–179.54)**	**48.3% (47.6%−49.2%)**	**2.4% (1.9% −3.0%)**

Data source: GBD/VLEG 2020 data [1]. Population Working age data [56]. UI: uncertainty interval. % Total Blindness or % Total MSVI was calculated as the quotient between the number of people with blindness or MSVI in the working age and the number of people with blindness or MSVI in all ages. % Working age population was calculated as the quotient between the number of people with blindness or MSVI in the working age and the working age population.

**Table 2:** Relative reduction in employment for people with vision loss (%).

GBD Super regions (bold) and regions	Relative reduction in employment among people with vision loss (%)
**High Income**	**32.12**
High-income Asia Pacific	26.70
Australasia	32.44
Western Europe	20.58
Southern Latin America	No data
High-income North America	43.46
	
**Central Europe, Eastern Europe, and Central Asia**	**22.50**
Central Asia	22.50
Central Europe	22.50
Eastern Europe	22.50
	
**Latin America and Caribbean**	No data
Caribbean	No data
Andean Latin America	No data
Central Latin America	No data
Tropical Latin America	No data
	
**North Africa and Middle East**	No data
	
**South Asia**	No data
	
**Southeast Asia, East Asia, and Oceania**	No data
Southeast Asia	No data
East Asia	No data
Oceania	No data
	
**Sub-Saharan Africa**	**28.85**
Central Sub-Saharan Africa	No data
Eastern Sub-Saharan Africa	No data
Southern Sub-Saharan Africa	No data
Western Sub-Saharan Africa	28.85
	
**Average**	**30.23**
	
**Number of countries with data**	**15**^a^

a Relative reduction in employment data was obtained from a literature search. We identified 11 studies [16], [17], [18], [19], [20], [21], [22], [23], [24], [25], [26] and 5 reportsce: [27], [28], [29], [30], [31] that provided employment reduction data for 15 countries and WHO Mortality Stratum regions, which we categorised into 8 GBD regions and 3 super regions

**Fig. 1:**
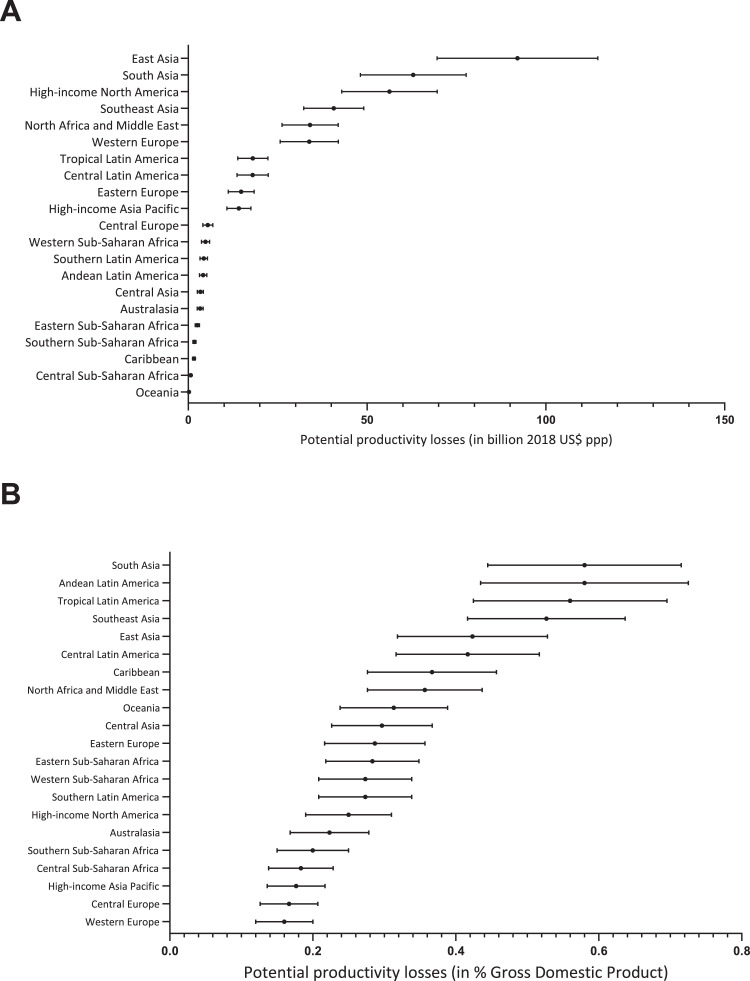
Productivity loss estimates using Gross Domestic Product (GDP) due to blindness and MSVI in the 21 Global Burden of Disease regions in 2018 (A) in billion US$ ppp (B) as a percentage of GDP.

Using GDP, we estimated the annual cost of potential productivity losses in 2018 was $410.7 billion ppp (range $322.1 billion to $518.7 billion ppp), of which $43.6 billion ppp (range $34.4 billion to $54.5 billion ppp) was due to blindness and $367.1 billion ppp (range $287.7 billion to $464.2billion ppp) was due to MSVI. This overall productivity loss amount represented 0.3% of the combined GDP of the 21 GBD regions in 2018. The regional estimates are presented in Table 3 and Fig. 1. East Asia (comprised of China and North Korea) was the region with the highest productivity loss estimates ($90.4 billion ppp; range $70.5 billion to $115.3 billion ppp) and Oceania the lowest ($0.2 billion ppp; range $0.1 billion to $0.2 billion ppp). Half (51%) of all global productivity losses were concentrated in three regions (East Asia, South Asia and High-income North America) primarily due to the high number of people with MSVI or blindness in East Asia and South Asia (50% of all people with vision loss in the working age population), and the high GDP per capita (supplementary Table 1), and high relative reduction in employment in High-income North America (Table 2). Productivity losses due to MSVI and blindness in South Asia represented 0.6% of the GDP in 2018 in this region, more than twice the impact found in North America (0.2% GDP). In contrast, Eastern Sub-Saharan Africa was the region with the lowest GDP per capita (approximately 4% of High-income North America) which led to the region accounting for only 0.6% of global potential productivity losses despite being home to 3% of people in the working age population with MSVI or blindness and having one of the highest employment to population ratios (supplementary Table 1). Productivity losses due to MSVI and blindness represented 0.3% of Eastern Sub-Saharan Africa GDP in 2018.

**Table 3:** Economic productivity losses by GBD region (US$ billion ppp, 2018).

GBD regions	GDP		GNI	
	Productivity Losses in US$ billion ppp (95%UI)	Productivity Losses in % GDP (95%UI)	Productivity Losses in US$ billion ppp (95%UI)	Productivity Losses in % GDP (95%UI)
High-income Asia Pacific	13.96 (10.9–17.62)	0.17 (0.14–0.22)	14.24 (11.12–17.97)	0.18 (0.14–0.22)
Australasia	3.28 (2.55–4.18)	0.22 (0.17–0.28)	3.18 (2.47–4.05)	0.21 (0.17–0.27)
Western Europe	33.34 (25.94–42.18)	0.16 (0.12–0.2)	33.53 (26.09–42.42)	0.16 (0.12–0.2)
Southern Latin America	4.27 (3.31–5.41)	0.27 (0.21–0.34)	4.10 (3.18–5.2)	0.26 (0.2–0.33)
High-income North America	55.51 (43.33–70)	0.25 (0.19–0.31)	56.19 (43.86–70.86)	0.25 (0.2–0.32)
Central Asia	3.33 (2.59–4.25)	0.29 (0.23–0.37)	3.15 (2.45–4.01)	0.28 (0.22–0.35)
Central Europe	5.37 (4.14–6.92)	0.16 (0.13–0.21)	5.18 (4–6.68)	0.16 (0.12–0.2)
Eastern Europe	14.54 (11.33–18.56)	0.28 (0.22–0.36)	14.18 (11.05–18.1)	0.27 (0.21–0.35)
Caribbean	1.59 (1.23–2.02)	0.36 (0.28–0.46)	1.39 (1.08–1.77)	0.32 (0.25–0.4)
Andean Latin America	4.12 (3.18–5.23)	0.57 (0.44–0.73)	3.95 (3.05–5.02)	0.55 (0.42–0.7)
Central Latin America	17.75 (13.77–22.51)	0.41 (0.32–0.52)	17.26 (13.39–21.88)	0.4 (0.31–0.5)
Tropical Latin America	17.83 (13.95–22.4)	0.55 (0.43–0.7)	17.55 (13.73–22.05)	0.54 (0.43–0.68)
North Africa and Middle East	33.56 (26.46–42.17)	0.35 (0.28–0.44)	33.48 (26.41–42.07)	0.35 (0.27–0.44)
South Asia	61.87 (48.68–78.22)	0.57 (0.45–0.72)	61.78 (48.61–78.11)	0.57 (0.45–0.72)
Southeast Asia	40.17 (32.57–49.33)	0.52 (0.42–0.64)	39.75 (32.23–48.82)	0.52 (0.42–0.63)
East Asia	90.39 (70.47–115.31)	0.42 (0.32–0.53)	90.06 (70.21–114.9)	0.41 (0.32–0.53)
Oceania	0.17 (0.13–0.21)	0.31 (0.24–0.39)	0.16 (0.13–0.21)	0.30 (0.23–0.38)
Central Sub-Saharan Africa	0.70 (0.54–0.9)	0.18 (0.14–0.23)	0.66 (0.51–0.85)	0.17 (0.13–0.22)
Eastern Sub-Saharan Africa	2.51 (1.97–3.15)	0.28 (0.22–0.35)	2.46 (1.94–3.1)	0.27 (0.21–0.34)
Southern Sub-Saharan Africa	1.71 (1.34–2.16)	0.20 (0.15–0.25)	1.66 (1.3–2.1)	0.19 (0.15–0.24)
Western Sub-Saharan Africa	4.75 (3.74–5.99)	0.27 (0.21–0.34)	4.56 (3.59–5.75)	0.26 (0.21–0.33)
**Total**	**410.70 (322.13 –518.74)**	**0.32 (0.25–0.41)**	**408.47 (320.38 –515.91)**	**0.32 (0.25–0.41)**

**Fig. 2:**
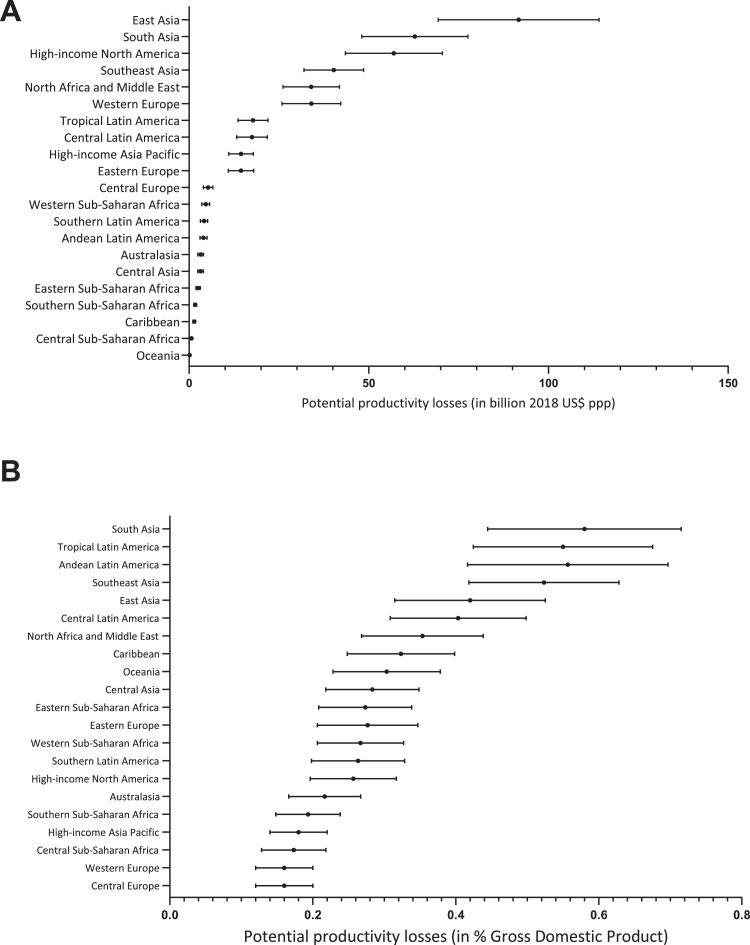
Productivity loss estimates due to blindness and MSVI derived from Gross National Income (GNI) in the 21 Global Burden of Disease regions in 2018 (A) in billion USD ppp, (B) as a percentage of GDP.

Using GNI, the overall productivity losses were estimated at $408.5 billion ppp (range $320.4 billion to $515.9 billion ppp), being $43.3 billion (range $34.2 billion to $54.1 billion ppp) due to blindness and $365.2 billion ppp (range $286.2 billion to $461.8 billion ppp) due to MSVI. At the global level, estimates using GNI were 0.5% lower than estimates using GDP and estimates were lower in 19 of the 21 GBD regions compared to when GDP was used (Table 3, Fig. 1 and Fig. 2). High-income Asia Pacific estimates had an increase of $0.3 billion ppp (2% increase compared with GDP estimates), while Caribbean estimates had a decrease of $0.2 billion ppp (12% reduction compared with GDP).

Using the Eurostat disability statistics, we estimated a relative reduction in employment of 19.5% at the global level (supplementary Table 3) compared to 30.2% used above (Table 2). Correspondingly, global productivity losses in this sensitivity analysis were 36% lower using both GDP ($262.6 billion ppp [range $205.8 billion to $331.9 billion ppp]) and GNI ($260.8 billion ppp [range $204.4 billion to $329.7 billion ppp]), supplementary Table 4. The two regions showing the largest reduction in productivity losses were High-income North America and North Africa and Middle East. Using disability weights as a proxy for productivity losses, we estimated a reduction in employment of 33.8% for blindness, 31.4% for severe VI and 8.9% for moderate VI. At the global level, estimates using disability weights reached $49.4 billion ppp (range $39.0 billion to $61.8 billion ppp) due to blindness, $194.8 billion ppp (range $152.6 billion to $246.4 billion ppp) due to severe VI and $55.2 billion ppp (range $43.3 billion to $69.8 billion ppp) due to moderate VI. The total productivity losses using disability weights were 27% lower compared to our main estimates when using GDP ($299.4 billion ppp [range $234.9 billion to $378.0 billion ppp]) and GNI ($297.5 billion ppp [range $233.4 billion to $375.7 billion ppp]).

## 4 Discussion

In 2020, 160.7 million people in the working age population were either blind or had MSVI, representing 3.3% of the global working age population. We combined these new MSVI and blindness prevalence data with updated employment gap and economic data, estimating the annual global productivity losses due to blindness and MSVI at $410.7 billion ppp (2018), or 0.3% of GDP in 2018. Our global estimate using GNI was very similar ($408.5 billion ppp), suggesting the estimates are not sensitive to differences between GDP and GNI at the global level.

We found limited data on the relative reduction in employment of people with vision loss, with a complete absence of data from some regions (table 2). However, our global estimate of 30.2% employment reduction is similar to a population-based survey conducted in 70 countries which reported that 21% of people of working age with ‘severe visual difficulties’ and 36% of people with ‘extreme visual difficulty’, who wanted to work, were not working [43]. As these data were not reported by country or region we were unable to include them in our model.

Previous studies have presented global and multi-country estimates of productivity loss, either for a specific group of regions or countries, or exclusively for blindness or specific eye conditions, such as refractive error or trachoma. One estimate of annual global productivity loss due to blindness was $26.8 billion ppp when we converted to 2018 $US ppp (supplementary Table 5) [34]. This amount is much lower than our estimate, largely because it did not include MSVI, but also because the probability of employment without vision loss was calculated as the product of the labour force participation rate and the unemployment rate, greatly reducing the number of people considered employable. A study that used a methodology similar to ours reported productivity losses for the WHO Regions of America A, Europe A, B1, B2 and C and West Pacific A1 and A2 of $193.3 billion ppp (when converted to 2018 $US ppp) [16]. These regions of mostly high-income countries roughly align with the regions in our study, High-income North America, Western Europe, Eastern Europe, Central Europe, Central Asia, Australasia and High-income Asia Pacific, for which we estimated losses of $129 billion ppp (GDP model).

Our GNI result ( $408.5 billion ppp) aligns with a recent global study that also used GNI, which estimated global productivity losses due to blindness and MSVI of $381 billion ppp, (converted to 2018 $US ppp) [35]. Compared to these two studies[16,35] that used similar methods, we drew on more extensive regional prevalence data which may have led to some of the difference in the estimates. Other reasons are that we assumed a more conservative employment gap, did not include premature mortality in our estimates, [16] and did not account for reduced wages [35] (more details provided in supplementary Table 5).

Sensitivity analysis demonstrated that our estimates were sensitive to changes in both prevalence and relative reduction in employment due to vision loss parameters. First, at a global level estimates varied from $322.1 billion to $518.7 billion (GDP model) when we used the upper and lower 95% uncertainty intervals of the crude prevalence of blindness and MSVI. Despite this uncertainty, these prevalence data are the most accurate and up-to-date information available [1]. The second sensitivity analysis used Eurostat disability data for the relative reduction in employment and found productivity losses to be 36% lower if relative reduction in employment due to vision loss is assumed to be equal to any other disability such as hearing, walking and communicating. There are examples, such as in Canada, where people with blindness had lower employment rates than people with any other disability [44]. However, this may reflect that employment rates vary according to the severity of disability, with people with more severe disability more likely to be out of the labour market [45,46,47]. We recognise that better data are needed for relative reduction in employment for people with vision loss, and believe that the data we used are more reliable than data for people with any disability. The third sensitivity analysis used disability weights reported by WHO as a proxy for relative reduction in employment and the subsequent productivity losses estimate decreased 27%. We believe these estimates should be interpreted with caution. These estimates assumed that people within the MSVI would be equally distributed across each of the moderate and severe VI categories. This assumption introduced additional uncertainty due to the lack of references to support this option. We also eliminated regional differences by applying the same disability weight for the 21 GBD regions regardless of development level. We used WHO disability weights instead of GBD disability weights [48] mainly because WHO methodology to calculate disability weights included multiple domains of health, functions, capacities and aspects of living [41]. The use of disability weights to estimate productivity losses has been considered less appropriate since a variety of health conditions have almost the same disability weight even if they may result in differing degrees of productivity losses [49]. Furthermore, GBD disability weights were based on a discrete choice comparisons of sequalae in terms of “who is healthier”, which may not sufficiently capture the impact of blindness and VI on everyday life, because even though blindness is highly undesirable, blind people are generally not considered sick or ill [41].

The strengths of this study include the development and use of a relatively simple formula to estimate potential productivity losses that can be easily replicated by countries and non-governmental organizations to evaluate the case for investing in interventions that increase employment opportunities for visually impaired individuals. Our estimates were based on the latest available data and used both GDP per capita and GNI per capita to enable comparability with previous estimates. These publicly available data are updated annually and are internationally standardized which increases the reliability of our estimates. We based our estimates of the relative reduction in employment due to vision loss on a literature review instead of following the assumptions made in previous studies such as assuming productivity losses being equal to disability weights[49], [50], [51] or assuming that a minimum of 70% of people with blindness and 30% of people with MSVI are not in paid employment [36,37].

Our analysis has several limitations. First, we were only able to find reports from 15 countries on which to base our estimates of the relative reduction in employment associated with vision impairment, and the severity of vision loss was rarely reported (Table 2). Moreover, prevalence data were available to us at the regional rather than country level. This lack of quality data from different countries, for different levels of severity, different age groups or by sex may increase uncertainty in our results. We performed a sensitivity analysis to study the impact of different data sources regarding the relative reduction in employment by using Eurostat statistics that include a wider range of countries and by estimating potential productivity losses separately for blindness and MSVI using disability weights. Estimates decreased in both sensitivity analyses, but more assumptions were necessary and therefore more uncertainty was introduced in both approaches compared to our primary estimate. Although we explored data insufficiency comprehensively, it is difficult to predict in what direction this data sparsity has affected the accuracy of our estimates either by overestimating or underestimating the productivity losses due to blindness and MSVI.

Second, there are several productivity loss components that we did not include in our estimates, such as those resulting from premature mortality, [16] absenteeism and presenteeism (reduced productivity in the work place), productivity losses of caregivers [52,53]. We also recognise that we have not included the productivity losses related to unpaid or informal labour activities. Our reason for not including these additional components such as, absenteeism, presenteeism and productivity losses of caregivers in our estimates is that reliable international data are currently lacking. We believe that excluding these elements is likely to have resulted in an underestimate of the overall magnitude of productivity losses due to blindness and MSVI.

Third, our estimates were limited to people under 65 years while other studies have assumed that the working age extends beyond 64 years [18]. Employment in people aged 65 years and older is largely influenced by the social protection and retirement pension systems in place at a national level, which vary greatly. For example, Western European countries generally have more favourable pension coverage and conditions than other countries and therefore people feel more secure to retire, with 8% of people aged 65–69 years remaining in paid employment [54]. In contrast, in Sub-Saharan Africa 39% of people in this age group continue to work, [54] perhaps because they feel less financially secure to retire. The relatively low employment participation rate among people aged 65 to 69 years in several regions means that a high proportion of the 39 million people in this age group who have MSVI or blindness are not employed (supplementary Table 6). We found a single report from Australia which reported a relative reduction in employment due to vision loss of 4.5% amongst people aged 65 years or older [18]. We recognise these data are limited, but had we included this age group in our model, our primary estimate of $410.7 billion ppp would not have been substantially different (i.e. 1.4% higher).

To improve future estimates of productivity losses, we need more studies reporting the employment status of people living with blindness and MSVI, particularly in low- and middle-income countries. Future research should investigate how different severity levels of vision impairment affect productivity losses and if there are relevant differences by gender, since traditionally women face more barriers finding and retaining employment [55]. Employment distribution by sector of activity and level of education are also important to characterize access, enablers and barriers to paid employment. Longitudinal studies rather than cross-sectional studies would increase our knowledge about changes of employment status over the course of an eye condition and identify possible baseline predictors of employment participation. These could be used in future models to improve productivity losses estimates in countries where only a few predictor variables are available. Comparative studies to evaluate national programs supporting employment in people with vision loss, availability of adaptive technology and societal perceptions of disability would help to understand which strategies are efficient and effective. A further extension could compare productivity losses from vision loss with those from other impairments and health conditions.

Furthermore, there is a need for more robust data on other components of productivity losses we had to omit, such as absenteeism and presenteeism, productivity losses of caregivers, and time lost from unpaid or informal labour activities as well as how these are associated with access and quality of health care. In particular, the relationship between vision impairment and unpaid labour, both in terms of its measurement and valuation, is an area that has received little attention. An increased understanding of this may allow it to be included in future economic studies. Without the inclusion of all of these components, estimates will continue to underestimate the magnitude of productivity losses. Increasing the number of studies reporting prevalence of vision impairment worldwide will also reduce uncertainty regarding prevalence and the subsequent productivity loss estimates. In this domain, prevalence data by country would allow for more detailed analysis of differences between countries and regions. Better data on employment status of people with vision impairment, other productivity loss components and more detailed prevalence data would provide more reliable information to analyse change over time and projections into the future that could aid strategic decision making. Finally, future estimates would benefit from more robust data for the 65–69 year-old age group, particularly in countries where the retirement age is increasing.

Employment is an important determinant of economic development, social inclusion and well-being for individuals, households, communities and nations. It supports financial independence, poverty reduction, physical and psychological health and quality of life [2]. Given the benefits of employment, the reduced employment levels amongst people with vision loss needs to be addressed. First, there are effective treatments for cataract and uncorrected refractive error, the leading causes of MSVI and blindness. Therefore, increasing access to treatment for these and other conditions should be a global priority to increase workforce participation and productivity gains. Second, for people whose vision cannot be restored, access to vision rehabilitation care and workplace adaptation should also be provided to help people with vision loss to stay in the labour market. Third, people in high-income countries, and with higher socioeconomic status within all countries, likely have better access to new technologies and vision aids to enable workforce participation. Solutions must be found to overcome these persistent inequities while developing and implementing policies that enable labour force participation by all who wish to pursue it. These policies could include incentive programmes to hire and support people with vision impairment, to adapt workplaces, and to promote equitable access to full and fair employment, promotion and career development plans. Through the assurance of fair employment and decent working conditions of people with vision loss, governments and the private sector can help eradicate poverty, alleviate social inequities, improve health, improve well-being, and increase economic productivity.

Our findings support the view that blindness and MSVI have a large economic impact worldwide. All world regions need to invest in increasing access to eye health services to prevent or treat avoidable vision loss, and to develop and deliver services and inclusive environments to enable visually impaired people to find and maintain employment. These actions would likely result in significant productivity gains.

## Authors’ contributions

MJB, APM, JC, KDF conceptualized and designed the study. APM collected data; RB provided VLEG prevalence data; APM and TB ran the model and verified all data. APM, TB, JC, JR, KDF, IJ, DM analysed and interpreted results. APM, JR, TB and JC wrote the first draft of the manuscript and MJB, JHZ, DM, IJ, BAT, BKS, HF, RB, KDF revised the manuscript. All authors reviewed and approved the final manuscript.

## Funding

MJB is supported by the Wellcome Trust (207472/Z/17/Z). JR's appointment at the University of Auckland is funded by the Buchanan Charitable Foundation, New Zealand. The *Lancet Global Health* Commission on Global Eye Health was supported by grants from The Queen Elizabeth Diamond Jubilee Trust, Moorfields Eye Charity (GR001061), NIHR Moorfields Biomedical Research Centre, The Wellcome Trust, Sightsavers, The Fred Hollows Foundation, The SEVA Foundation, The British Council for the Prevention of Blindness and Christian Blind Mission. The funders had no role in the design, conduct, data analysis of the study, or writing of the manuscript.

## Data sharing statement

This modelling study used published or publicly available data. The data used and the sources are described in this Article and the appendix.

## Declaration of Competing Interest

MJB reports research support grants as the Principal Investigator from The Queen Elizabeth Diamond Jubilee Trust Moorfields Eye Charity (GR001061), NIHR Moorfields Biomedical Research Centre The Wellcome Trust (20190426_PH2), Sightsavers, The Fred Hollows Foundation, SEVA Foundation, British Council for the Prevention of Blindness, and the Christian Blind Mission, paid to specifically support the work of the Commission to the LSHTM. JR's role at the University of Auckland is funded by the Buchanan Charitable Trust, New Zealand. DM is employed by The Fred Hollow Foundation that both receives donations and funds programs in eye health. IJ is an employee of Sightsavers. The Lancet Global Health Commission on Global Eye Health was supported by grants from a number of organisations, including Sightsavers. RRAB reports grants from Brien Holden Vision, Foundation Théa, Lions Club International, Gates Foundation, Sightsavers, and University of Heidelberg. All other authors have nothing to disclose.

## Appendix Supplementary materials

Image, application 1
