# Supplementary material for: Global economic productivity losses from vision impairment and blindness
Source: eClinicalMedicine. 2021 Apr 26;35:100852. doi: 10.1016/j.eclinm.2021.100852 (PMC8093883; doi:10.1016/j.eclinm.2021.100852)
Supplement: Supplementary file 1 [file mmc1.docx]

**Supplementary appendix**

1. **Employment to population ratio**

The employment-to-population ratio is defined as the proportion of a country’s working-age population that is employed. A high ratio means that a large proportion of a country’s population is employed, while a low ratio means that a large share of the population is not involved directly in labour market-related activities, because they are either unemployed or out of the labour force for other reasons (e.g. studying, early retired).

Employment-to-population ratio for each of the 21 GBD regions was calculated as a weighted average of employment to population ratio of each country^1^ included in each region with total population of each country being the weights.

$$\frac{\sum_{n=nr countries} \left( Empl.ratio x {Tot. pop}_{count a} \right)+\left( {Empl ratio}_{count b} x {Tot. pop}_{count b} \right)+\ldots\left( {Empl ratio}_{count n} x {Tot Pop}_{Count n} \right)}{{\sum_{n=nr countries in each subregion} Tot.Pop}_{country a}+ {Tot. Pop}_{country b}+\ldots{Tot.Pop}_{Country n}}$$

Tot.Pop designates Total Population; nr= number of countries included in each region; Empl. ratio=Employment to population ratio

Eleven countries had no information related to employment and therefore were not included in this weighted average: Seychelles, Antigua and Barbuda, Dominica, Grenada, Syrian Arab Republic, Kiribati, Marshall Islands, Micronesia, Djibouti, Somalia and Andorra

1. **Gross Domestic Product (GDP)/Gross National Income (GNI)**

GDP per capita ppp and GNI per capita ppp for each of the 21 GBD regions were calculated as a weighted average of GDP per capita^2^ or GNI per capita^3^ of each country included in each region with total population of each country being the weights.

The weighted Average of GDP per capita ppp of all the countries included in each region were calculated as the quotient between:

- Sum of the product of GDP per capita of each country and total population (Tot.Pop) of each country included in each region; and
- Sum of total population of each country included in each region

GDP per capita per sub region = Weighted Average of GDP per capita ppp of all the countries included in each region =$\frac{\sum_{n=nr countries} \left( {GDP pc}_{country a} x {Tot. pop}_{country a} \right)+\left( {GDP pc}_{country b} x {Tot. pop}_{country b} \right)+\ldots\left( {GDP pc}_{country n} x {Tot Pop}_{Country n} \right)}{{\sum_{n=nr countries} Tot.Pop}_{country a}+ {Tot. Pop}_{country b}+\ldots{Tot.Pop}_{Country n}}$

Tot.Pop designates Total Population; n= number of countries included in each subregion; pc=per capita

The weighted Average of GNI per capita ppp was calculated in the same way.

There was no data for GDP or GNI per capita ppp in Cuba, Korea Dem. People’s Republic, Syrian Arab Republic, Djibouti, Somalia and Andorra therefore these countries were not included in this computation.

Employment to population ratio, GDP and GNI for the 21 GBD regions are reported at Supplementary Table 1.

**Supplementary Table 1: Employment to population ratio (%), Gross Domestic Product (GDP) and Gross National Income (GNI) in in 2018 US Dollar purchasing power parity ($US ppp)**

| **GDB Regions** | Employment population ratio (%),weighted Average* | GDP per capita, PPP ($US ppp), weighted Average** | GNI per capita, PPP ($US ppp), weighted Average |
| --- | --- | --- | --- |
| High-income Asia Pacific | 59.82 | 43,931.14 | 44,804.84 |
| Australasia | 62.39 | 49,920.62 | 48,310.19 |
| Western Europe | 53.85 | 47,396.43 | 47,661.81 |
| Southern Latin America | 55.88 | 22,059.34 | 21,190.63 |
| High-income North America | 59.76 | 61,302.54 | 62,051.88 |
| Central Asia | 59.81 | 13,639.55 | 12,889.09 |
| Central Europe | 52.80 | 28,932.16 | 27,941.54 |
| Eastern Europe | 56.57 | 22,839.47 | 22,268.70 |
| Caribbean | 41.99 | 14,053.39 | 12,327.34 |
| Andean Latin America | 70.84 | 12,429.64 | 11,927.74 |
| Central Latin America | 60.07 | 16,765.21 | 16,295.69 |
| Tropical Latin America | 56.29 | 16,016.16 | 15,765.47 |
| North Africa, Middle East | 44.55 | 19,390.17 | 19,347.32 |
| South Asia | 51.75 | 7,111.99 | 7,101.73 |
| Southeast Asia | 64.92 | 12,363.58 | 12,235.47 |
| East Asia | 64.50 | 18,236.61 | 18,170.00 |
| Oceania | 48.35 | 4,744.20 | 4,614.76 |
| Central Sub-Saharan Africa | 63.55 | 2,952.21 | 2,767.45 |
| Eastern Sub-Saharan Africa | 75.20 | 2,385.85 | 2,347.68 |
| Southern Sub-Saharan Africa | 48.68 | 11,521.06 | 11,187.40 |
| Western Sub-Saharan Africa | 58.36 | 4,352.62 | 4,182.71 |

*data missing for 11 countries [Seychelles, Antigua and Barbuda, Dominica, Grenada, Syrian Arab Republic, Kiribati, Marshall Islands, Micronesia, Djibouti, Somalia and Andorra ]

**data missing for 6 countries [Cuba, Korea Dem. People’s Republic, Syrian Arab Republic, Djibouti, Somalia and Andorra]

For the majority of countries, the latest available data were reported for the year 2018. That was not the case for:

1. Cyprus where the latest available data for GNI per capita PPP and GDP per capita PPP was from the year 2017;
2. Iran where the latest available data for GNI per capita PPP and GDP per capita PPP and GDP, PPP were from the year 2017
3. Algeria where the latest available data for GNI per capita PPP was from the year 2017
4. Eritrea where the latest available data for total population, GNI per capita PPP and GDP per capita PPP and GDP PPP was from the year 2011;
5. Venezuela where the latest available data for GNI per capita PPP and GDP per capita PPP was from the year 2014; and GDP PPP was from the year 2011
6. Yemen where the latest available data for GNI per capita PPP was from the year 2014 and GDP PPP was from the year 2013
7. **Relative reduction in employment for people with vision loss**

An estimate of the relative reduction in employment for people with vision impairment was identified through a literature search. We sought studies or reports from any country published since the year 2000 that reported the employment status and/or employment gap in people with vision impairment. We identified 11 published studies^4-17^ and 5 reports ^18-21^ briefly described in Supplementary Table 2.

The employment gap for each region and super region was calculated as the weighted average employment gap (with the total population of each country being the weight) of the countries included in each region or super region that reported data.

When estimating productivity losses by region we used the super region average whenever there was no data for a specific region. If there were no data for either region or super region the average of all super region was used. These values are reported in Table 2 of the manuscript.

**Supplementary Table 2: Employment gaps due to vision loss identified in the literature**

| **Reference** | **Country and population** | **GBD Region** | **VI definition** | **Remarks** | **Relative reduction in employment** |
| --- | --- | --- | --- | --- | --- |
| Gordois et al and Access Economics^4,5^ | Australia 40-65 years | Australasia | VA < 6/12 | This study reported employment reduction in people with VI in 7 regions using data from 4 countries: Australia, Canada, Japan and United Kingdom. Employment gaps for Australia and Japan were applied to regions Western Pacific region -A1 and Western Pacific region -A2, respectively. Employment reduction from the United Kingdom was applied to regions Europe A, B1, B2, and C. Employment reduction for Americas region-A was derived from a Canadian study. Employment reduction was calculated by comparing employment rates in people with and without VI. | Western Pacific region A1 44% |
|  | Japan  Working age | H.I Asia Pacific | VA of one eye is 0.02 or less and VA of the other eye is 0.6 or less |  | Western Pacific region A2 26.7% |
|  | Canada  Working age | H.I North America | Seeing disability |  | Americas region A 47% |
|  | United Kingdom Working age | Western Europe | Seeing disability |  | Europe region A, B, C 22.5%. |
| Access Economics and Pezzullo ^6,7^ | United Kingdom Working age | Western Europe | Seeing disability | This study reported employment reduction in people with VI in the United Kingdom in 4 age groups. Employment rates by age group and gender derived from Office of National Statistics (ONS) (2007b) and the employment gap found when comparing employment rates for people without disability and without seeing difficulties and people with seeing difficulties reported by Institute for Employment Studies (IES) (2008) were used to derived employment gap due to VI by age groups. This study has used the average gap across all levels of visual acuity | 18 - 24 years (15.7%)  25-34 years (19.5%)  35-49 years (19.9%)  50 -64 years (17.2%) |
| Taylor et al ^8,9^ | Australia  40 – 64 years,  ≥65 years | Australasia | Not reported | This study reported the impact of vision loss on employment rates in 2 age groups based on a previous report (Access Economics ^9^). Employment rates in these 2 age groups were compared in people with and without VI. These differences were then adjusted by age. | 40 - 64 years (27.5%)  ≥65 years (4.5%) |
| Access Economics ^10^ | Australia  40 – 64 years,  ≥65 years | Australasia | Not reported | This study reported the impact of vision loss on employment rates in 2 age groups based on a previous report (Access Economics ^9^). Employment rates in these 2 age groups were compared in people with and without VI. These differences were then adjusted by age. | 40 - 64 years (27.5%)  ≥65 years (4.5%) |
| Rein et al ^11^ | United States  40 – 64 years | H.I North America | Seeing disability | This study reported reduced labour force participation rates for people with VI and blindness aged 40 to 64 years old. Labour force participation rates published from the 1997 Survey Income and Program Participation from people reporting having some difficulty in seeing words or letters (to indicate visual impairment) and people reporting having severe difficulty in seeing words or letters to indicate blindness. | 40 - 64 years with VI (41%)  40 - 64 years with blindness (55%) |
| Gordon et al ^12^ | Canada  Working age | H.I North America | Seeing disability | This study reported employment reduction in people with vision loss. Employment rates published from the Participation and Activity Limitation Survey (2001) were compared with national employment rates for each age-gender group. | 18% |
| Adio et al ^13^ | Nigeria  16 – 64 years | Western sub-Saharan Africa | Not reported | This study supported their productivity losses estimates based in a previous study (Cook 2008) which reported labour force participation rates in people with and without blindness. | 30% |
| Schakel et al ^14^ | Netherland  ≥18 years | Western Europe | WHO VI and Blindness definition | This study collected and reported work status (paid employment), absence from work, reduced efficiency at work (presenteeism), and productivity losses for unpaid work (i.e., substitution for domestic tasks) in a sample of 480 individuals. Data were collected through the use of Labour Questionnaire (SF-HLQ). Number and percentage of people in paid employment, absenteeism and self-reported work efficiency was reported in individuals with and without vision impairment | 57% |
| Marques et al ^15^ | Portugal  17-64 years | Western Europe | WHO VI and Blindness definition | This study collected and reported work status (paid employment) and absence from work in a sample of 546 individuals. Data were collected from a validated and pretested questionnaire. Number and percentage of people in paid employment and absenteeism were reported in individuals with and without vision impairment. Employment rate found in this sample was compared with employment rates of the active  population in the country. | 36% |
| Access Economics ^16^ | Australia  40 – 64 years  ≥65 years | Australasia | Not reported | This study reported the impact of vision loss on employment rates in 2 age groups based on a previous report (Access Economics^9^). Employment rates in these 2 age groups were compared in people with and without VI. These differences were then adjusted by age. | 40 - 64 years old (27.5%)  65 or over (4.5%) |
| Onabulu et al ^17^ | Nigeria  17-68 years | Western sub-Saharan Africa | VA < 3/60 | This study collected and reported work status (paid employment), education and occupation in a sample of 66 blind individuals. Data were collected using a pretested questionnaire. Employment status on people with blindness were split in 5 categories: self-employed, government employment, private organization employment, unemployed, students. Employment rate was calculated considering government and private organization employment. | 27.3% |
| American foundation for the blind ^18^ | United States  Working age | H.I North America | Not reported | The American foundation for the blind reported employment statistics for people with VI and blindness based on the 2017 American Community Survey. Employment rates of people who were blind or visually impaired were compared with individuals without disability. Employment data was spilt by education level. | 35% |
| EuroBlind organization ^19^ | Not specified  Working age | Western Europe | WHO VI and Blindness definition | The European blind organization reported unemployment rates of blind and partially sighted persons of working age without providing details on how these data were collected or obtained. | 75% |
| RNIB – Royal National Institute for the Blind | United Kingdom Working age | Western Europe | Seeing disability | This study reported employment reduction in people with VI in in the United Kingdom in 4 age groups. Employment rates by age group derived from Office of National Statistics (2007b) and the employment gap found when comparing employment rates for people not disabled and without seeing difficulties and people with seeing difficulties reported by Institute for Employment Studies (IES (2008) were used to derived the employment gap due to VI by age group. This study used the average gap across all levels of visual acuity | 18 - 24 years (15.7%)  25-34 years (19.5%)  35-49 years (19.9%)  50 -64 years (17.2%) |
| US Bureau of Labor Statistics ^20^ | United States  16-64 years | H.I North America | Seeing disability | The U.S. Bureau of Labor Statistics reported employment data in people with disabilities. This information was obtained from the Current Population Survey (CPS), a sample survey (n= 60,000 individuals) that provides statistics on employment and unemployment in the United States. Individuals or household members aged 15 years and above were classified as having a disability if they responded yes to 5 questions including “Is anyone blind or does anyone have serious difficulty seeing even when wearing glasses?". Employment population ratio for people with disability were compared between people with and without disabilities. | 43.6% |
| Blind Foundation^21^ | Canada  Working age | H.I North America | Seeing disability | A survey developed by CNIB Foundation, Vision Australia and the Blind Foundation of New Zealand reported employment in people with sight loss. This was a first-of-its-kind multinational survey conducted in 2018 and included a sample of 1,205 Canadians, 362 Australians and 357 New Zealanders. Full time employment was reported by educational level, severity of sight loss, onset of sight loss, and industry. Results reported a strong correlation between education and employment status but no correlation between severity of sight loss and full-time employment status. | 28% |
|  | Australia  Working age | Australasia |  |  | 24% |
|  | New Zealand  18-64 years | Australasia |  |  | 33% |

H.I – High Income; VA – Visual Acuity; VI – Vision Impairment; CNIB- Canadian National Institute for the Blind

1. **Eurostat Disability statistics**

To assess uncertainty due to the relative reduction in employment data, we used Eurostat disability statistics data. Eurostat disability statistics^22^ reported employment reduction data from 31 countries included in 4 regions and 3 super regions for people reporting disabilities in basic activities defined as “activity diﬃculty such as sight, hearing, walking and communicating”.

We grouped this information by region and super region calculating a weighted average reduction in employment for the countries in the region/super region for which there was data. We used the total population of each /region country as the weight. For regions and super regions without data we followed the same approach as described to relative reduction in employment of people with MSVI or blindness. These values are detailed in the following supplementary table:

**Supplementary Table 3: Relative reduction in employment for people reporting disability in basic activities (%)**

| **GDB Super Regions and Regions** | **Eurostat disability statistics** |
| --- | --- |
| **High Income** | **18.9** |
| High-income Asia Pacific | No data |
| Australasia | No data |
| Western Europe | 18.9 |
| Southern Latin America | No data |
| High-income North America | No data |
| **Central Europe, Eastern Europe, and Central Asia** | **30.0** |
| Central Asia | No data |
| Central Europe | 30.7 |
| Eastern Europe | 18.5 |
| **Latin America and Caribbean** | No data |
| Caribbean | No data |
| Andean Latin America | No data |
| Central Latin America | No data |
| Tropical Latin America | No data |
| **North Africa, Middle East** | **9.90** |
| **South Asia** | No data |
| **Southeast Asia, East Asia, and Oceania** | No data |
| Southeast Asia | No data |
| East Asia | No data |
| Oceania | No data |
| **Sub-Saharan Africa** | **No data** |
| Central Sub-Saharan Africa | No data |
| Eastern Sub-Saharan Africa | No data |
| Southern Sub-Saharan Africa | No data |
| Western Sub-Saharan Africa | No data |
|  |  |
| **Number of countries with data** | **31** |
| **Average** | **19.55** |

**Supplementary Table 4: Annual economic productivity losses in US$ billion ppp, 2018 by GBD regions, estimated using GDP and GNI.**

| **GBD regions** | **GDP** | **GNI** |
| --- | --- | --- |
|  | **Productivity Losses in billion US$ ppp (95%UI)** | **Productivity Losses in billion US$ ppp (95%UI)** |
| High-income Asia Pacific | 9.88 (7.72-12.47) | 10.08 (7.87-12.72) |
| Australasia | 1.91 (1.49-2.44) | 1.85 (1.44-2.36) |
| Western Europe | 31.68 (24.65-40.08) | 31.85 (24.79-40.30) |
| Southern Latin America | 2.51 (1.95-3.18) | 2.41(1.87-3.06) |
| High-income North America | 24.13(18.84-30.43) | 24.43 (19.07-30.81) |
| Central Asia | 4.44 (3.46-5.66) | 4.20 (3.27-5.35) |
| Central Europe | 7.33 (5.65-9.45) | 7.08 (5.46-9.12) |
| Eastern Europe | 11.94 (9.31-15.25) | 11.64 (9.07-14.87) |
| Caribbean | 1.03 (0.79-1.31) | 0.90 (0.70-1.15) |
| Andean Latin America | 2.66 (2.06-3.38) | 2.56 (1.97-3.25) |
| Central Latin America | 11.48 (8.91-14.55) | 11.16 (8.66-14.15) |
| Tropical Latin America | 11.53 (9.02-14.48) | 11.35 (8.88-14.26) |
| North Africa and Middle East | 10.99 (8.67-13.81) | 10.96 (8.65-13.78) |
| South Asia | 40.01 (31.48-50.58) | 39.95 (31.43-50.51) |
| Southeast Asia | 25.97 (21.06-31.90) | 25.70 (20.84-31.57) |
| East Asia | 58.45 (45.57-74.57) | 58.24 (45.40-74.30) |
| Oceania | 0.11 (0.08-0.14) | 0.11 (0.08-0.14) |
| Central Sub-Saharan Africa | 0.48 (0.37-0.61) | 0.45 (0.34-0.57) |
| Eastern Sub-Saharan Africa | 1.70 (1.34-2.14) | 1.67 (1.32-2.10) |
| Southern Sub-Saharan Africa | 1.16 (0.91-1.46) | 1.12 (0.88-1.42) |
| Western Sub-Saharan Africa | 3.22 (2.53-4.06) | 3.09 (2.43-3.90) |
| **Total** | **262.60 (205.83-331.95)** | **260.79 (204.42-329.66)** |

1. **Countries listed by GBD Super Region and Region**

Our results have been reported by Global Burden of Disease (GBD) super region (7) and region (21)^23^, as below:

**High-income**

**Australasia:** Australia, New Zealand

**High-income Asia Pacific:** Brunei Darussalam, Japan, South Korea, Singapore

**High-income North America:** Canada, United States

**Southern Latin America:** Argentina, Chile, Uruguay

**Western Europe**: Andorra, Austria, Belgium, Cyprus, Denmark, Finland, France, Germany, Greece, Iceland, Ireland, Israel, Italy, Luxembourg, Malta, Netherlands, Norway, Portugal, Spain, Sweden, Switzerland, United Kingdom

**Central Europe, Eastern Europe and Central Asia**

**Central Europe:** Albania, Bosnia and Herzegovina, Bulgaria, Croatia, Czech Republic, Hungary, Macedonia, Montenegro, Poland, Romania, Serbia, Slovakia, Slovenia

**Eastern Europe:** Belarus, Estonia, Latvia, Lithuania, Moldova, Russian Federation, Ukraine

**Central Asia:** Armenia, Azerbaijan, Georgia, Kazakhstan, Kyrgyzstan, Mongolia, Tajikistan, Turkmenistan, Uzbekistan

**Latin America and Caribbean**

**Andean Latin America:** Bolivia, Ecuador, Peru

**Caribbean:** Antigua and Barbuda, Bahamas, Barbados, Belize, Cuba, Dominica, Dominican Republic, Grenada, Guyana, Haiti, Jamaica, Puerto Rico, Saint Lucia, Saint Vincent and the Grenadines, Suriname, Trinidad and Tobago

**Central Latin America:** Colombia, Costa Rica, El Salvador, Guatemala, Honduras, Mexico, Nicaragua, Panama, Venezuela

**Tropical Latin America:** Brazil, Paraguay

**North Africa and Middle East**

**North Africa and Middle East:** Afghanistan, Algeria, Bahrain, Egypt, Iran, Iraq, Jordan, Kuwait, Lebanon, Libyan Arab Jamahiriya, Morocco, Occupied Palestinian Territory, Oman, Qatar, Saudi Arabia, Sudan, Syrian Arab Republic, Tunisia, Turkey, United Arab Emirates, Yemen

**South Asia**

**South Asia:** Bangladesh, Bhutan, India, Nepal, Pakistan

**Southeast Asia, East Asia and Oceania**

**East Asia:** China, Dem. People´s Republic of Korea, Taiwan

**Southeast Asia:** Cambodia, Indonesia, Lao People´s Democratic Republic, Malaysia, Maldives, Mauritius, Myanmar, Philippines, Seychelles, Sri Lanka, Thailand, Timor-Leste, Vietnam

**Oceania:** Fiji, Kiribati, Marshall Islands, Micronesia, Papua New Guinea, Samoa, Solomon Islands, Tonga, Vanuatu

**Sub-Saharan Africa**

**Central Sub-Saharan Africa:** Angola, Central African Republic, Congo, Democratic Republic of the Congo, Equatorial Guinea, Gabon

**East Sub-Saharan Africa:** Burundi, Comoros, Djibouti, Eritrea, Ethiopia, Kenya, Madagascar, Malawi, Mozambique, Rwanda, Somalia, Uganda, United Republic of Tanzania, Zambia

**Southern Sub-Saharan Africa:** Botswana, Lesotho, Namibia, South Africa, Swaziland, Zimbabwe

**West Sub-Saharan Africa:** Benin, Burkina Faso, Côte d´Ivoire, Cameroon, Cape Verde, Chad, Gambia, Ghana, Guinea, Guinea-Bissau, Liberia, Mali, Mauritania, Niger, Nigeria, São Tomé and Príncipe, Senegal, Sierra Leone, Togo

1. **Comparison of global productivity losses from vision impairment**

**Supplementary Table 5: Comparison of global productivity losses from vision impairment**

|  | **Marques et al (present study)** | **Gordois 2012^4^** | **Bastawrous 2019^24^** | **Frick 2003^25^** |
| --- | --- | --- | --- | --- |
| Definition of vision impairment | Blindness is defined as presenting distance visual acuity <3/60 in the better eye and MSVI as presenting distance visual acuity of <6/18 to 3/60 in the better eye. | Blindness was defined as visual acuity <6/60 and moderate VI as visual acuity <6/18 to 6/60 | Blindness was defined as presenting visual acuity <20/400 in the better-seeing eye; MSVI was defined as presenting visual acuity <20/60 to 20/400 in the better-seeing eye | Blindness was defined as visual acuity less than 3/60 in the better eye; Low vision was defined as visual acuity less than 6/18 to 3/60 in the better eye. |
| Year of cost data | 2018 | 2010 | 2017 | 2000 |
| Prevalence data source | Crude prevalence and 95% uncertainty intervals for blindness and MSVI in people within working age, by GBD region. Data source: these data were specifically calculated by GBD Vision Loss Expert Group for 2020 for this analysis.^26^ | Prevalence data using WHO global VI estimates.^27,28^ | Prevalence data for blindness and MSVI by country were obtained from the GBD Vision Loss Expert Group 2015 estimates.^29^ It was assumed that 40% and 50% of the blind and MSVI population were of working age respectively. | Prevalence data for blindness and low vision by World Development region and age group were obtained for 1995.^30^ |
| Regions of report | Global | WHO ´developed` regions Americas region -A, Europe region -A, Europe region B1, Europe region -B2, Europe region-C, Western Pacific region-A1, and Western P-A2. It was assumed that only in developed countries a loss in productivity due to VI is expected to represent a real cost to an economy | Global | Global |
| Working age population | 15 to 64 years | 15 to 64 years | 15 to 64 years | 15 to 64 years |
| Probability of Employment in the general population | It is assumed that someone with VI is just as likely to have been employed in the absence of their VI condition. This likelihood is measured using the employment to population ratio. | It was assumed that someone with VI is just as likely to be employed in the absence of their VI condition. This likelihood was expressed using employment to population ratio | Not included | Labour force participation rate and unemployment rate were used to calculate probability of employment |
| Relative reduction in employment due to vision impairment | Unemployment in people with VI was derived from a literature review. Reports from 15 countries were used to estimate unemployment in people with VI by GBD region using a weighted average (total population used as weight) | Under– or unemployment in people with VI was derived from a literature review. Reports from 5 countries was used to extrapolate data for each subregion. | Under-or unemployment in people with VI was assumed to be equal to 50% for people with blindness or MSVI | Under- or unemployment in people with VI was assumed to be equal to disability weight: 60% for people with blindness and 24.5% for people with VI |
| Value of production loss equal to | GDP & GNI per capita | GDP per capita | GNI per capita | GDP per capita |
| Productivity losses components included estimates | Reduced employment participation | Reduced employment participation and premature mortality | Reduced employment participation and reduced wages (based on USA data only) | Reduced employment participation |
| Productivity loss estimate  Converted to 2018 USD ppp | Annual Global productivity losses were estimated to be 410.7 billion USD ppp (range $322.1 - $518.7 billion) in GDP model and $408.5 billion ppp (range $320.4 - $515.9 billion) in GNI model | Annual Global productivity losses from blindness and moderate VI in the 7 ´developed` WHO regions was estimated at 193.36 billion USD PPP including 1 billion due to premature mortality | Global productivity losses from blindness and VI were estimated to be ~381 billion USD ppp in 2020. Estimated to rise cumulatively to 19.4 trillion USD PPP in 2050. | Annual Global productivity losses ranged from 26.8 billion USD ppp (for blindness) to 59.4 billion USD ppp of productivity loss (blindness and low vision) |
| VI = Vision Impairment; MSVI = Moderate Severe Vision Impairment; PPP = Purchasing Power Parity; GDP = Gross Domestic Product; GNI= Gross National Income; GBD = Global Burden of Disease; WHO = World Health Organization; | | | | |

1. **Number of people age 65 to 69 years old with blindness or moderate to severe vision impairment in the 21 Global Burden of Disease regions in 2020**

### Supplementary Table 6: Number of people aged 65 to 69 years with blindness or moderate to severe vision impairment across the 21 Global Burden of Disease regions in 2020.

| **GBD regions** | **Number of people aged 65 to 69 years with blindness** | **Number of people aged 65 to 69 years with moderate to severe vision impairment** |
| --- | --- | --- |
|  | **Number in millions**  **(95%UI)** | **Number in millions**  **(95%UI)** |
| High-income Asia Pacific | 0.04 (0.03-0.05) | 0.4 (0.32.0.49) |
| Australasia | 0.01 (0.00-0.01) | 0.05 (0.04-0.06) |
| Western Europe | 0.11 (0.08-0.14) | 1.01 (0.8-1.25) |
| Southern Latin America | 0.01 (0.01-0.02) | 0.16 (0.12-0.19) |
| High-income North America | 0.06 (0.05-0.07) | 0.58 (0.47-0.71) |
| Central Asia | 0.03 (0.02-0.04) | 0.31 (0.25-0.38) |
| Central Europe | 0.04 (0.03-0.05) | 0.53 (0.42-0.66) |
| Eastern Europe | 0.09 (0.07-0.11) | 1.49 (1.19-1.84) |
| Caribbean | 0.02 (0.02-0.03) | 0.14 (0.11-0.17) |
| Andean Latin America | 0.03 (0.02-0.04) | 0.23 (0.19-0.29) |
| Central Latin America | 0.11 (0.09-0.14) | 0.82 (0.66-1.01) |
| Tropical Latin America | 0.18 (0.14-0.22) | 0.86 (0.69-1.05) |
| North Africa and Middle East | 0.38 (0.28-0.50) | 2.15 (1.74-2.59) |
| South Asia | 1.5 (1.17-1.89) | 12.99 (10.71-15.48) |
| Southeast Asia | 0.7 (0.54-0.9) | 3.40 (2.89-3.97) |
| East Asia | 1.23 (0.95-1.62) | 7.77 (6.15-9.60) |
| Oceania | 0.00 (0.00-0.01) | 0.04 (0.03-0.05) |
| Central Sub-Saharan Africa | 0.03 (0.02-0.04) | 0.18 (0.15-0.22) |
| Eastern Sub-Saharan Africa | 0.20 (0.15-0.26) | 0.61 (0.50-0.73) |
| Southern Sub-Saharan Africa | 0.06 (0.03-0.07) | 0.15 (0.12-0.18) |
| Western Sub-Saharan Africa | 0.25 (0.19-0.33) | 0.96 (0.78-1.16) |
| **Total** | **5.07 (3.98-6.47)** | **34.83 (28.34-42.02)** |

Data source: GBD/VLEG 2020 data.^23^ UI: uncertainty interval

The calculation of the annual cost of potential productivity losses in this age group included: (1) the number of people with blindness or MSVI aged 65 to 69 years old (shown above); (2) the Labour force participation rate of people aged 65 years and over by region^31^ ; 3) the relative reduction in employment for people with vision loss aged 65 years or older^8^; (4) per capita Gross Domestic Product (GDP) or Gross National Income (GNI) for 2018.

**References**

1. World Bank. World Development Indicators: Employment to population ratio, 15+, total (%) (modeled ILO estimate). Washington: The World Bank Group, 2020.

2. World Bank. World Development Indicators: GDP per capita, PPP (current international $). Washington: The World Bank Group, 2020.

3. World Bank. World Development Indicators: GNI per capita, PPP (current international $). Washington: The World Bank Group, 2020.

4. Gordois A, Cutler H, Pezzullo L, et al. An estimation of the worldwide economic and health burden of visual impairment. *Global public health* 2012;**7**:465-81.

5. Economics A. The Global Economic Cost of Visual Impairment. Melbourne, Australia: Access Economics, 2010.

6. Economics A. The economic impact of partial sight and blindness in the UK adult population. Melbourne,Australia: Royal National Institute of blind people, 2009.

7. Pezzullo L, Streatfeild J, Simkiss P, Shickle D. The economic impact of sight loss and blindness in the UK adult population. *BMC health services research* 2018;**18**:63.

8. Taylor HR, Pezzullo ML, Keeffe JE. The economic impact and cost of visual impairment in Australia. *The British journal of ophthalmology* 2006;**90**:272-5.

9. Economics A. Clear Insight: The Economic Impact and Cost of Vision Loss in Australia. Melbourne, Australia: Centre Eye Research Australia, University of Melbourne, 2004.

10. Economics A. Clear focus: The economic impact of of vision loss in Australia in 2009: Melbourne, Australia, 2010.

11. Rein DB, Zhang P, Wirth KE, et al. The economic burden of major adult visual disorders in the United States. *Archives of ophthalmology (Chicago, Ill : 1960)* 2006;**124**:1754-60.

12. Gordon KD, Cruess AF, Bellan L, Mitchell S, Pezzullo ML. The cost of vision loss in Canada. 1. Methodology. *Can J Ophthalmol* 2011;**46**:310-4.

13. Adio AO, Onua AA. Economic burden of glaucoma in Rivers State, Nigeria. *Clinical ophthalmology (Auckland, NZ)* 2012;**6**:2023-31.

14. Schakel W, van der Aa HPA, Bode C, Hulshof CTJ, van Rens GHMB, van Nispen RMA. The Economic Burden of Visual Impairment and Comorbid Fatigue: A Cost-of-Illness Study (From a Societal Perspective). *Investigative ophthalmology & visual science* 2018;**59**:1916-23.

15. Marques AP, Macedo AF, Lima Ramos P, et al. Productivity Losses and Their Explanatory Factors Amongst People with Impaired Vision. *Ophthalmic epidemiology* 2019:1-15.

16. Economics A. The Economic Impact of Diabetic Eye Disease :a dynamic economic model. Melbourne, Australia: Centre Eye Research Australia, University of Melbourne, 2008.

17. Onabolu OO, Bodunde OT, Ajibode AH, Otulana TO, Ebonhor M, Daniel OJ. Rehabilitation and paid employment for blind people in a low income country. *Journal of Advances in Medicine and Medical Research* 2018;**25** 1-9.

18. American Foundation for the Blind. Reviewing the Disability Employment Research on People who are Blind or Visually Impaired: Key Takeaways. 2020. <https://www.afb.org/research-and-initiatives/employment/reviewing-disability-employment-research-people-blind-visually>;

19. EuroBlind Organization. About blindness and partial sight: facts and figures. 2020. <http://www.euroblind.org/about-blindness-and-partial-sight/facts-and-figures>;

20. U.S. Bureau of Labor Statistics. Persons with a disability: Labor force characteristics - 2019 Washington, 2020.

21. Blind Foundation. Blind people significantly under employed around the world. 2018. <https://www.scoop.co.nz/stories/PO1811/S00173/blind-people-significantly-under-employed-around-the-world.htm>;

22. Eurostat. Employment rate of people by type of disability, sex and age. v363-20200615-acc6e-ESTAT_LINUX_PROD DATA-EXPLORER_PRODmanaged14: v3.6.3-20200615-acc6e-ESTAT_LINUX_PROD DATA-EXPLORER_PRODmanaged14, 2020.

23. Bourne R, Adelson J, Flaxman S, et al. Trends in prevalence of Blindness and distance and near Vision Impairment over 30 Years and contribution to The Global Burden of Disease in 2020. *The Lancet Global Health* 2020.

24. Bastawrous A, Suni AV. Thirty Year Projected Magnitude (to 2050) of Near and Distance Vision Impairment and the Economic Impact if Existing Solutions are Implemented Globally. *Ophthalmic Epidemiol* 2019:1-6.

25. Frick KD, Foster A. The magnitude and cost of global blindness: an increasing problem that can be alleviated. *Am J Ophthalmol* 2003;**135**:471-6.

26. Bourne R. Magnitude, temporal trends, and projections of the global prevalence of blindness and distance and near vision impairment: a systematic review and meta-analysis - VLEG/GBD 2020. *SUBMITTED* 2020.

27. Resnikoff S, Pascolini D, Etya'ale D, et al. Global data on visual impairment in the year 2002. *Bull World Health Organ* 2004;**82**:844-51.

28. Resnikoff S, Pascolini D, Mariotti SP, Pokharel GP. Global magnitude of visual impairment caused by uncorrected refractive errors in 2004. *Bull World Health Organ* 2008;**86**:63-70.

29. Bourne RRA, Flaxman SR, Braithwaite T, et al. Magnitude, temporal trends, and projections of the global prevalence of blindness and distance and near vision impairment: a systematic review and meta-analysis. *Lancet Glob Health* 2017;**5**:e888-e97.

30. Thylefors B, Negrel AD, Pararajasegaram R, Dadzie KY. Global data on blindness. *Bull World Health Organ* 1995;**73**:115-21.

31. ILO. What about seniors?: a quick analysis of the situation of older persons in the labour market. In: (ILO) ILO, ed. Genève, Switzerland: ILO Statistics, 2018.
